# Report on Practical Medicine

**Published:** 1851-07

**Authors:** 


### ARTICLE III.

### REPORT ON PRACTICAL MEDICINE,

The Report of the Committee on Practical Medicine to the American Medical Association for the year 1850, by Prof. Austin Flint, of Buffalo, as printed and laid before the meeting at Charleston, lies before us. If the plan of printing before presentation had been adopted by the committees generally in reference to their Reports, it would have done away with the supposed necessity for abolishing the standing committees—a measure, of the propriety of which, we have very serious doubts.

The report, as the profession had good grounds from the reputation of its author to expect, is a very well drawn up and able document. Although with some of the views of the author we cannot agree, we are willing to award to the report the mead of praise so justly its due, for being an authentic and reliable, as well as a well-selected and well-digested collection of facts from among the American improvements, for the last year in practical medicine. But our limits will not allow us to give an abstract of its contents, for which reason we lay it aside for future notice.

